# Release-aware, SAS-equivalent Elixhauser comorbidity scoring in R: cross-implementation validation and application to Texas inpatient discharges

**DOI:** 10.64898/2026.10.01.26364551

**Published:** 2026-10-05

**Authors:** Minh Tran, Dong Pei

## Abstract

The Elixhauser comorbidity measures are among the most widely used risk adjusters in administrative health data. Their reference implementation, published by the Agency for Healthcare Research and Quality (AHRQ) as SAS programs, has eleven annual releases in two families, one screening pre-existing conditions on the present-on-admission (POA) indicator and the earlier one on Medicare Severity Diagnosis-Related Group, and no implementation of every release existed outside SAS. We developed ecsr10, an open-source R package covering all eleven releases of both families, with a browser-based application over the same functions, and validated it against AHRQ’s own SAS programs over 40,027,114 value-level comparisons with zero disagreements. Two existing open-source reimplementations scored on the same dataset showed reproducible defects. We then used the package to quantify two choices AHRQ’s software leaves to the analyst and studies seldom report, the release and the handling of diagnoses not present on admission, on the Texas Inpatient Public Use Data File, 2016 Q1–2019 Q4 (8,585,244 adult discharges from 726 hospitals), scoring one predefined cohort under every release and pre-existing-condition setting. POA handling dominated: disabling POA changed the comorbidity profile of 56.7% of admissions, and a POA-naive mortality index scored higher on discrimination (area under the curve 0.807 versus 0.789; difference 0.0183, 95% confidence interval 0.0161–0.0204) by counting in-hospital complications as pre-existing disease. POA reporting was bimodal across hospitals, so pooled hospital comparisons on such a file partly compare documentation practice. Because the cohort predates every release compared, the release contrast is a lower bound: the release chosen between v2022.1 and v2026.1 changed at least one comorbidity flag for 0.0036% of admissions, yet a single revision of ten mortality weights changed the comorbidity index for 42.4% of them. An exactly validated implementation makes such choices measurable; release, POA handling and the implementation used should be reported as study characteristics.

**Author summary:**

Hospital discharge records are routinely used to compare hospitals, adjust for how sick patients were on arrival, and predict outcomes. Much of that work rests on comorbidity measures maintained by a United States federal agency, whose reference software exists only in SAS and has changed eleven times. We built a free package that reproduces the agency’s programs exactly in every version, checked it against the agency’s own programs with no disagreement, and found that two existing free reimplementations do not. We then used it to score 8.6 million Texas hospital admissions every way the agency’s software allows, to ask which of the choices studies rarely report change conclusions. Far more consequential than the annual version was whether the software is told which diagnoses patients had on arrival. Ignoring that information counts hospital complications as pre-existing illness. It changed the comorbidity profile of more than half of admissions, and made a mortality model look more accurate while measuring something else. Hospitals also differed sharply in whether they recorded that information. The annual version barely moved the comorbidity flags, yet one revision of the scoring weights changed the risk score for 42% of admissions.

## Full Text Availability

The license terms selected by the author(s) for this preprint version do not permit archiving in PMC. The full text is available from the preprint server.

